# Diagnostic Accuracy of MRI for Detecting Cervical Invasion in Patients with Endometrial Carcinoma: A Meta-Analysis

**DOI:** 10.7150/jca.52797

**Published:** 2021-01-01

**Authors:** Qiu Bi, Guoli Bi, Junna Wang, Jie Zhang, Hongliang Li, Xiarong Gong, Lixiang Ren, Kunhua Wu

**Affiliations:** 1 Department of MRI, the First People' s Hospital of Yunnan Province, the Affiliated Hospital of Kunming University of Science and Technology, No. 157 Jinbi Road, Kunming 650032, Yunnan, China.; 2 Center of Infectious Diseases, West China Hospital of Sichuan University, No. 37 Guoxue Lane, Wuhou District, Chengdu 610041, Sichuan, China.

**Keywords:** endometrial carcinoma, magnetic resonance imaging, diffusion weighted imaging, cervical invasion, meta-analysis

## Abstract

**Objectives:** To evaluate the diagnostic accuracy of magnetic resonance imaging (MRI) in the preoperative assessment of cervical invasion and to analyse the influence of different imaging protocols in patients with endometrial carcinoma.

**Methods:** An extensive search of articles about MRI for assessing cervical invasion in patients with endometrial carcinoma was performed on PubMed, Embase, Web of Science, Cochrane Library, and Clinical Trials from January 2000 to July 2020. Two reviewers independently evaluated the methodological quality of each study by using the Quality Assessment of Diagnostic Accuracy Studies-2 (QUADAS-2). Diagnostic accuracy results and additional useful information were extracted. The pooled estimation data was obtained by statistical analysis.

**Results:** A total of 42 eligible studies were included in the meta-analysis. Significant evidence of heterogeneity was found for detecting cervical invasion (*I^2^* = 74.1%, *P* = 0.00 for sensitivity and* I^2^* = 56.2%, *P* = 0.00 for specificity). The pooled sensitivity and specificity of MRI were 0.58 and 0.95 respectively. The use of higher field strength (3.0 T) demonstrated higher pooled sensitivity (0.74). Using diffusion weighted imaging (DWI) alone presented higher pooled sensitivity (0.86) than using other sequences. The studies that used dynamic contrast-enhanced MRI (DCE-MRI) alone showed higher sensitivity (0.80) and specificity (0.96) than those that used T2-weighted imaging (T2WI) alone.

**Conclusions:** MRI shows high specificity for detecting cervical infiltration in endometrial carcinoma. Using DWI or a 3.0-T device may improve the pooled sensitivity. DCE-MRI demonstrates higher pooled sensitivity and specificity than T2WI.

## Introduction

Endometrial carcinoma is one of the most common gynaecological malignancies 1. Cervical invasion is an important prognostic factor for endometrial carcinoma and is associated with a higher risk of lymph node metastases 2,3. Hysterectomy and bilateral salpingo-oophorectomy are the primary treatments for endometrial carcinoma 4. However, in patients with cervical infiltration, radical hysterectomy or preoperative radiotherapy with bilateral salpingo-oophorectomy and bilateral pelvic-para-aortic lymphadenectomy may be necessary 4. Consequently, it is important to preoperatively evaluate the cervical involvement when planning treatments.

Magnetic resonance imaging (MRI) is widely used to detect cervical invasion in endometrial carcinoma and is also more accurate than hysteroscopy 5 and endocervical curettage 6. MRI has no ionizing radiation and has high soft-tissue resolution for the uterus and cervix. Therefore, in contrast to computed tomography 7 and transvaginal sonography 8, MRI is considered to be the optimal imaging modality for the preoperative assessment of cervical invasion 9. With the development of functional MRI imaging, diffusion weighted imaging (DWI) and dynamic contrast-enhanced MRI (DCE-MRI) are increasingly applied to detect cervical infiltration in endometrial carcinoma 10-14. Many studies have investigated the accuracy of MRI for detecting cervical invasion 6,12-31. These studies differ in the MR pulse sequences, magnetic field strength, and number of patients, so the results are diverse. This leads to an ongoing dispute about the accuracy of MRI and the best imaging protocol for evaluating cervical involvement of endometrial carcinoma.

The purpose of the present study was to evaluate the diagnostic accuracy of MRI for detecting cervical invasion and to analyse the effects of different imaging protocols in patients with endometrial carcinoma.

## Materials and Methods

### Literature search

We performed this meta-analysis according to the Preferred Reporting Items for Systematic Reviews-Diagnostic Test Accuracy (PRISMA-DTA) guidelines 32. A comprehensive literature search of articles pertaining to the accuracy of cervical invasion using MRI in endometrial carcinoma was performed by using the following keywords (including subject word and random word): “endometrial neoplasms”, “magnetic resonance imaging”, and “cervical”. Two authors (G.B., a radiologist with 20 years of experience and Q.B., a radiologist with 5 years of experience) independently conducted the searches on PubMed, Embase, Web of Science, Cochrane Library, and Clinical Trials from January 2000 to May 2020 for English language articles on human subjects. To identify possible missing citations, the reference lists of the relevant articles were manually searched.

### Study selection

The two authors who performed the literature search independently reviewed all the titles, abstracts, and full texts to identify potentially eligible articles. Studies meeting the following criteria were included if: (a) the accuracy for the detection of cervical invasion in endometrial carcinoma was evaluated; (b) the histopathological results after surgical resection were used as the reference standard; and (c) sufficient information was presented to allow for reconstruction of 2 × 2 tables. When the data or patient cohorts overlapped in the included studies, we selected the article with the largest number of patients.

### Data extraction and processing

The data on the diagnostic accuracy results and additional useful information in the original studies were collected by two researchers (Q.B. and J.W.) who had 5 years of independent experience in data extraction for diagnostic studies. In cases where the researchers had discrepancies, a consensus was reached after discussion with each other. For each study, the following information was extracted: author name, year of publication, nation, patient age, sample size, number of observers, study design, patient recruitment, blinded to reference, magnetic field, manufacturer, sequences of observing cervical infiltration, depth of cervical invasion, interval between MRI and pathology, and the true-positive, true-negative, false-positive, and false-negative values of MRI in detecting cervical invasion in patients with endometrial carcinoma. When two or more observers were in the same study, the most experienced observer was selected; if their experience was not reported, the first observer was selected. The most contemporary MRI scan (e.g., DWI before DCE-MRI) was preferred when different MR pulse sequences were reported in the same study. When the accuracy of any cervical invasion and stromal invasion was reported separately, the latter was preferred.

### Assessment of data quality

Quality assessment was conducted by the Quality Assessment of Diagnostic Accuracy Studies-2 33 (QUADAS-2) by two investigators (G.B. and J.Z., a radiologist with 20 years of experience in pelvic imaging) independently. Any disagreements were resolved by discussion with each other. The QUADAS-2 form is composed of four domains: patient selection (assessing methods of patient selection), index test (assessing the index test and how it was conducted and interpreted), reference standard (assessing the reference standard and how it was conducted and interpreted), and flow and timing (assessing any patients who did not receive the index test and/or reference standard or who were excluded from the 2 × 2 table).

### Statistical analysis

The analyses were performed by using Review Manager 5.3 (The Nordic Cochrane Centre, Copenhagen, Denmark), MetaDisc 1.4 (Ramón y Cajal Hospital, Madrid, Spain), and Stata 15.1 (StataCorp, Texas, USA). The threshold effect was assessed by the Spearman correlation coefficient between the logit of sensitivity and the logit of (1-specificity) 34. *P* values < 0.05 were indicated that the threshold effect existed 34. Heterogeneity for sensitivity and specificity was explored by using the inconsistency index (*I*^2^ value) in forest plots 35. *I*^2^ values ≥ 50.0% are considered to indicate substantial heterogeneity 35. A fixed-effects model was used to summarize the overall pooled diagnostic results if homogeneity existed. A random-effects model was utilized if heterogeneity existed. Summary receiver operating characteristic (sROC) curves and the area under the curve were used to elucidate the relationship between the sensitivity and specificity. If heterogeneity existed, meta-regression was performed to assess covariates. Several relevant covariates were as follows: patient age (≥ 60 years or < 60 years), magnetic field (1.5 T or 3.0 T), MR pulse sequences, design (prospective or retrospective), blind to reference (yes or unknown), depth of cervical infiltration (stromal invasion or any cervical invasion), and appropriate interval between MRI and pathology (yes or unknown). Sensitivity analyses were performed on the basis of those potential influencing factors of heterogeneity. The publication bias was assessed by using Deeks'funnel plot with *P* values < 0.05 36.

## Results

### Literature search and data extraction

**Figure 1:**
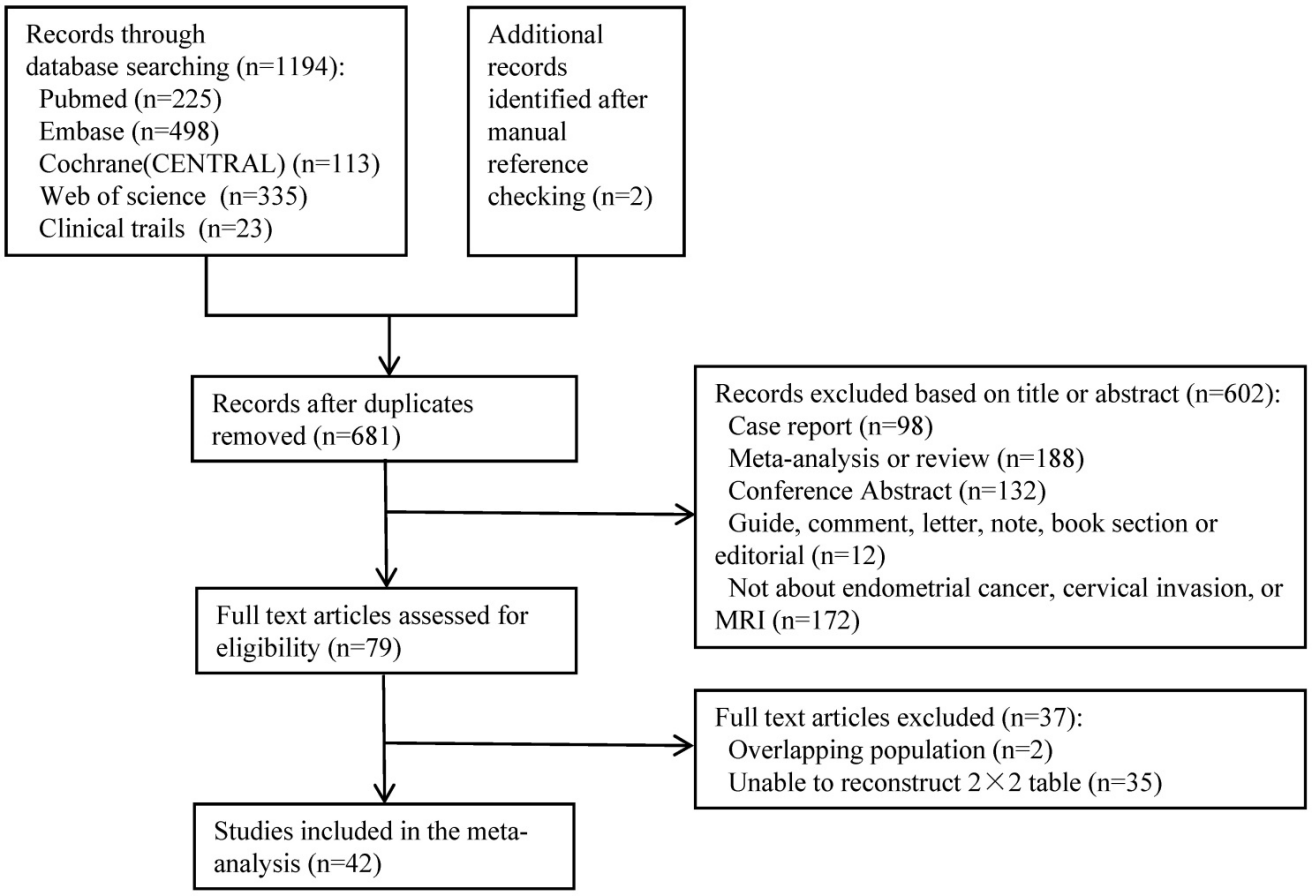
Flowchart of the study selection process.

The detailed flowchart summarizing the literature search and selection is given in **Figure 1.** A total of 1194 records from January 2000 to July 2020 of English language articles on human subjects were found. Two additional records were identified after manual reference checking. After duplicates were removed, 681 unique citations remained. Based on the screening of titles and abstracts, 602 studies were excluded. The full text of 79 studies was reviewed, and 42 eligible studies involving 4196 patients were included in this meta-analysis. Among these 4196 patients, 739 were confirmed to have cervical invasion by surgical pathology. The mean prevalence of endometrial carcinoma with cervical invasion was 17.6% (range, 4.5%-39.5%). The details of the principal characteristics of every included study are summarized in **Table 1.**

### Quality assessment and publication bias

**Figure 2:**
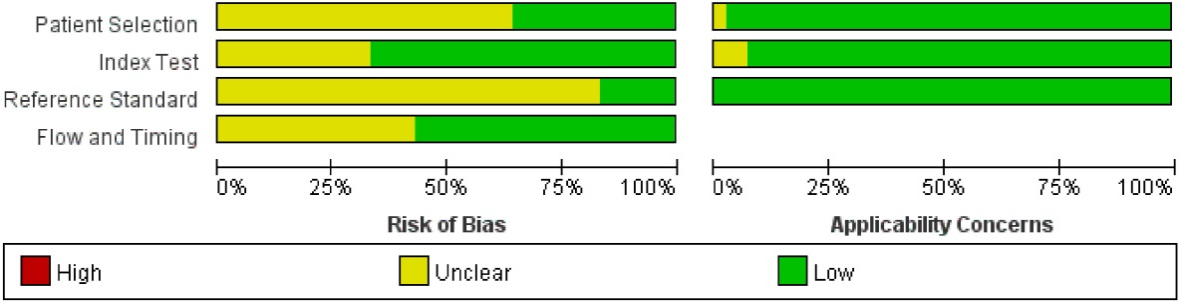
Histogram plot of the Quality Assessment of Diagnostic Accuracy Studies-2 (QUADAS-2) scores of the methodological study quality.

**Figure 2** shows the methodological quality graph of the evaluation of the risk of bias and applicability concerns of the selected studies, according to the evaluation based on QUADAS-2. Regarding the risk of bias and the patient selection domain, 14 studies explicitly reported that the patients were consecutive 10,12,13,16,21,23,25-28,31,37-39, and the remaining 28 studies only reported the start and end times of patient recruitment 5,6,8,11,14,15,17-20,22,24,29,30,40-53. Concerning the index test domain, 6 studies did not report that histopathology was blinded from the MRI findings 5,22,23,27,40,45. Eleven studies did not present the threshold for defining cervical invasion 5,8,15,17,22,37,38,45,48,51,52. For the reference standard domain, only 7 studies explicitly stated that pathology results were blinded to MRI findings 18,20,24,26,31,38,41, the remaining 35 studies lacked information for the reference domain. In relation to the flow and timing domain, twenty-four studies reported an appropriate interval between the MRI and pathological examination 5,6,8,10,12-16,18-21,23,24,26,30,31,38,39,41,44,45,50, and the remaining 18 studies did not report the interval. All studies applied pathological evaluation of the removed uterus except for one study where the evaluation was not reported 22.

**Figure 3:**
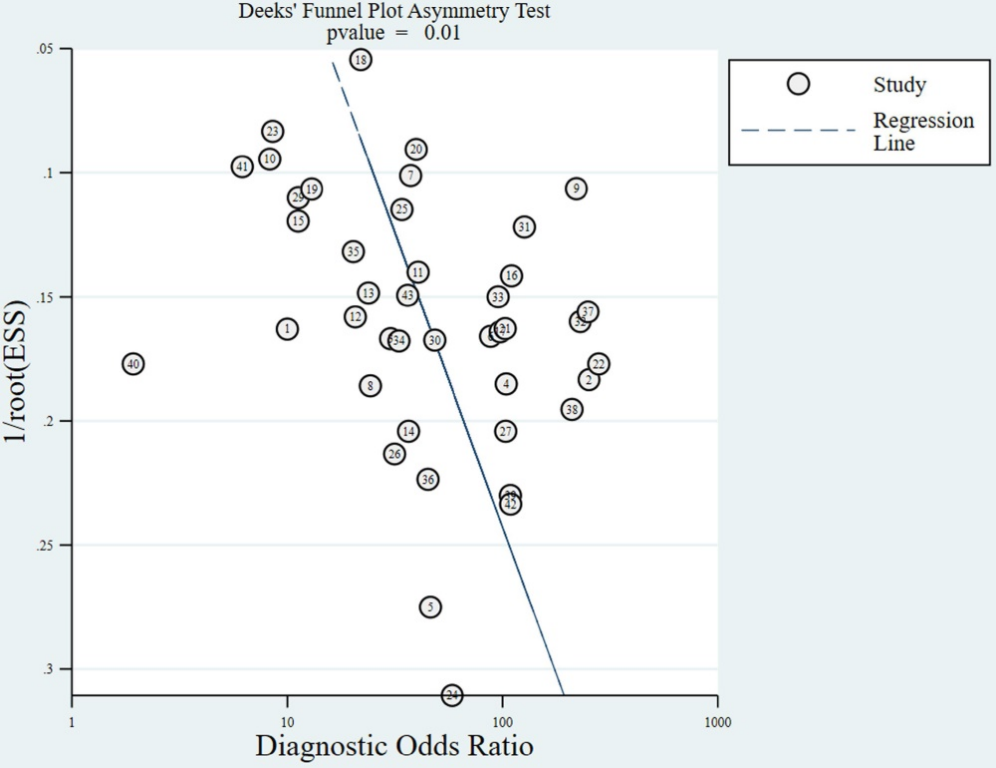
Deeks'funnel plot for evaluating cervical invasion in endometrial carcinoma. A value of *P*<0.05 was considered to indicate significant publication bias. The numbers in circles represent the study numbers. ESS, effective sample size.

The slope coefficients for the Deeks'funnel plot for MRI in assessing cervical invasion in endometrial carcinoma are presented in **Figure 3.** Publication bias was detected in the funnel plots for the diagnosis of cervical invasion (*P* = 0.01).

### Diagnostic accuracy

**Figure 4:**
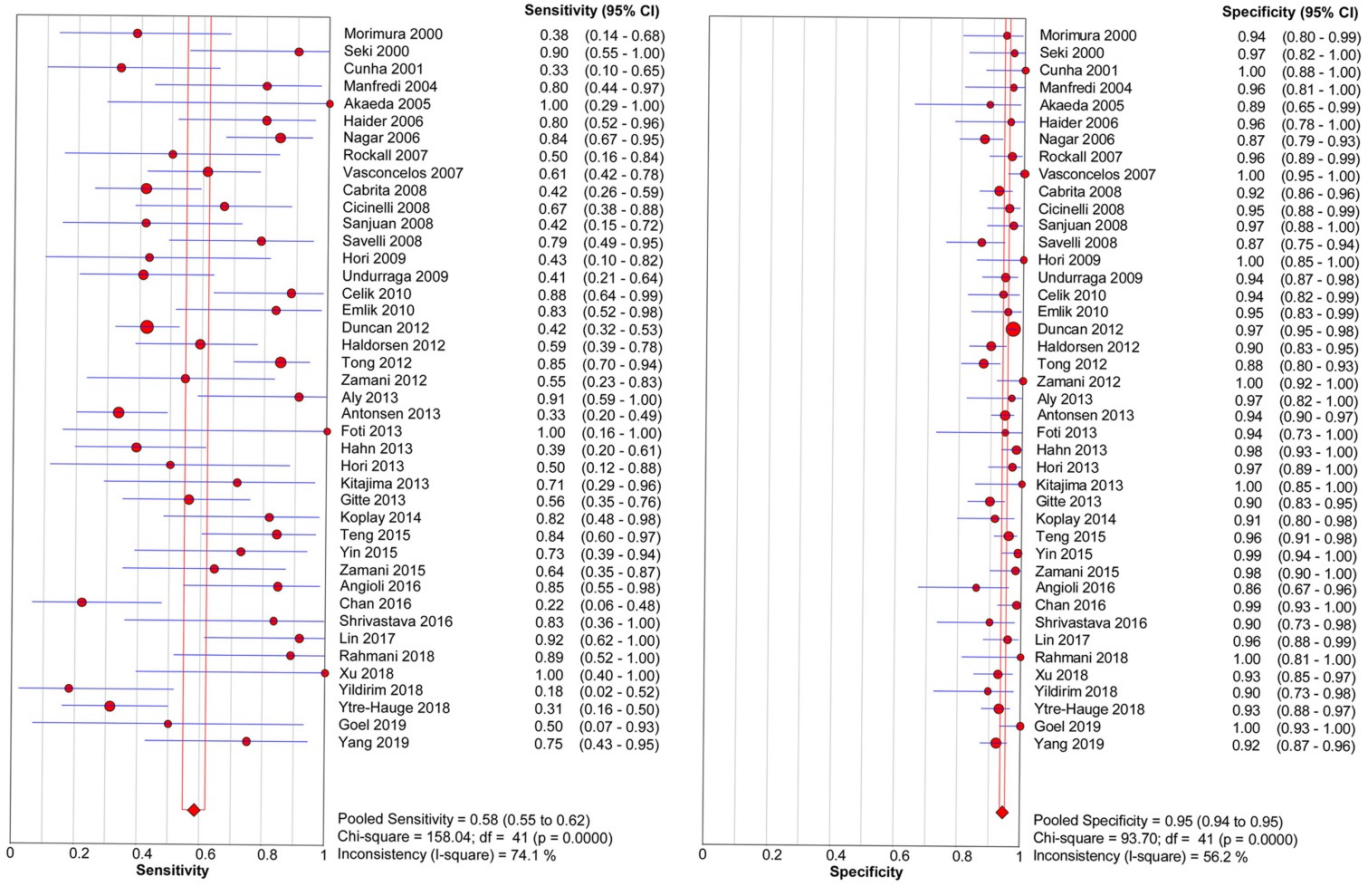
Forest plots show the pooled sensitivity and specificity of MRI for detecting cervical invasion in endometrial carcinoma. *I2* values ≥ 50.0% are considered to indicate substantial heterogeneity in each study. CI, confidence interval.

**Figure 5:**
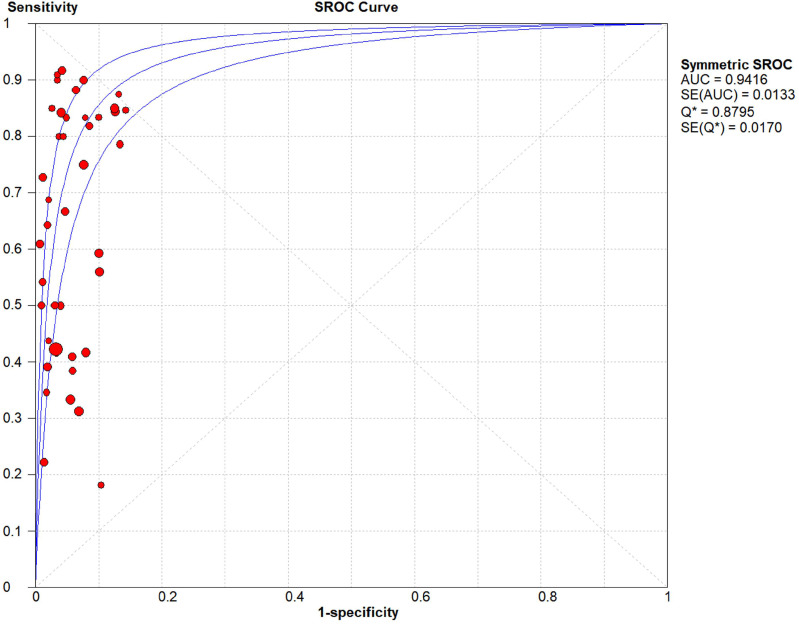
Summary receiver operating characteristic (sROC) curves of MRI for detecting cervical invasion in endometrial carcinoma. AUC, area under the curve.

**Figure 6:**
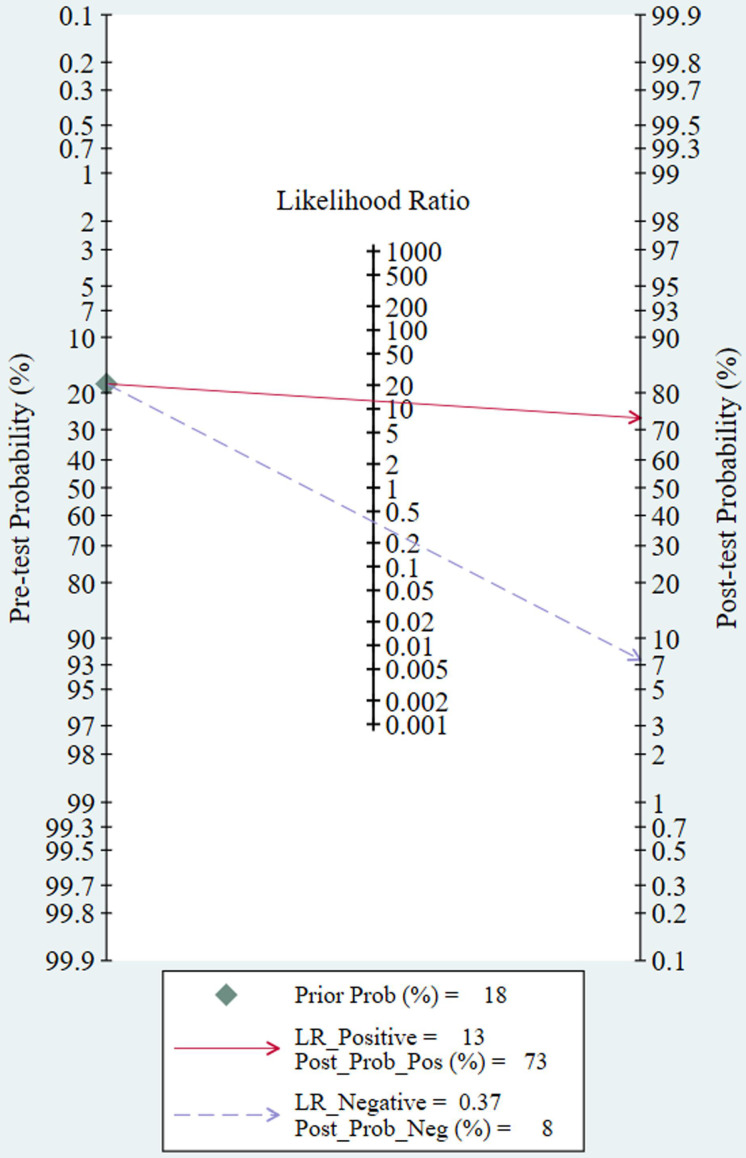
The Fagan nomogram shows the pre-test probability, positive post-test probability, and negative post-test probability of MRI for assessing cervical involvement in endometrial carcinoma. LR, likelihood ratio; Prob, probability.

The threshold effect did not exist for detecting cervical invasion (Spearman correlation coefficient = -0.282, *P* = 0.070). **Figure 4** shows the forest plots of the sensitivity and specificity of MRI for detecting cervical invasion, which showed significant evidence of heterogeneity (*I*^2^ = 74.1%, *P* = 0.000 for sensitivity and *I*^2^ = 56.2%, *P* = 0.000 for specificity). The pooled sensitivity, specificity, positive likelihood ratio, negative likelihood ratio, and diagnostic odds ratios for the diagnostic accuracy of MRI in detecting cervical invasion were 0.58 (95% confidence interval [CI] 0.55-0.62), 0.95 (95% CI 0.94-0.95), 9.37 (95% CI 7.78-11.28), 0.43 (95% CI 0.36-0.51), and 29.68 (95% CI 21.16-41.63), respectively. On the basis of sROC (**Figure 5**), the area under the curve was 0.94. Fagan nomograms showed that the pre-test probability of cervical invasion was 18%, and the corresponding positive post-test probability and negative post-test probability were 73% and 8%, respectively (**Figure 6**).

### Meta-regression and sensitivity analyses

Meta-regression showed that patient age, magnetic field, MR pulse sequences, design, blinding method, depth of cervical infiltration, and interval between MRI and pathology did not explain the heterogeneity observed for sensitivity and specificity (**Table 2**).

**Figure 7:**
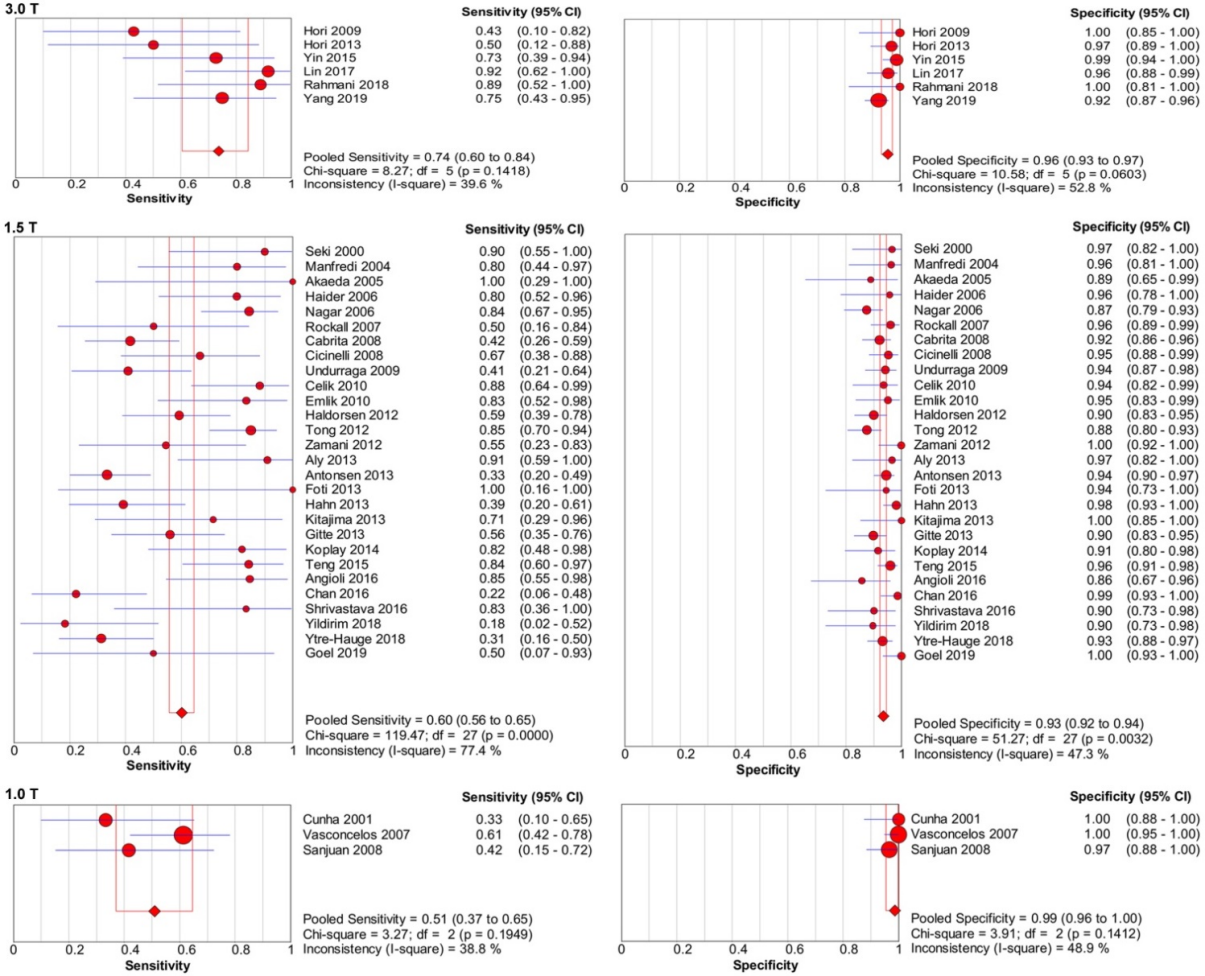
Forest plots of pooled sensitivity and specificity of using a 3.0-T device, 1.5-T device, and 1.0-T device for assessing cervical involvement in endometrial carcinoma. CI, confidence interval.

**Figure 8:**
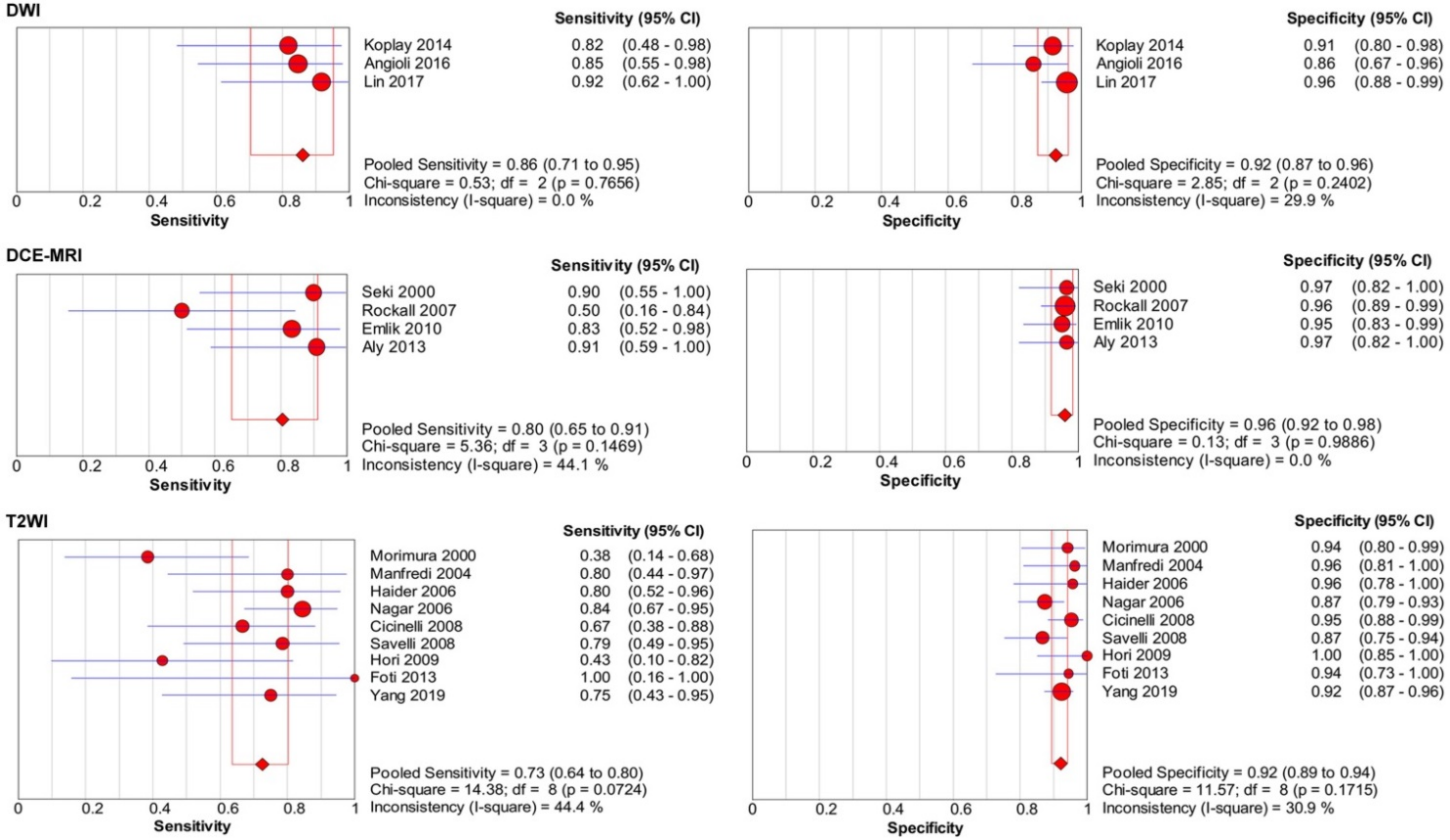
Forest plots of pooled sensitivity and specificity of diffusion weighted imaging (DWI), dynamic contrast-enhanced MRI (DCE-MRI), and T2-weighted imaging (T2WI) for assessing cervical involvement in endometrial carcinoma. CI, confidence interval.

**Table 3** presents the results of the sensitivity analyses performed for the different subgroups. Overall, several differences were observed for the sensitivity and specificity estimates in sensitivity analyses, and the forest plots of sensitivity and specificity are presented in **Figure 7-8.** Studies with higher field strength (3.0 T) had higher pooled sensitivity (0.74; 95% CI: 0.60-0.84) than studies with a 1.5-T device (0.60; 95% CI: 0.56-0.65) or 1.0-T device (0.51; 95% CI: 0.37-0.65). Additionally the higher the field strength was, the higher the pooled sensitivity. However, the pooled specificity was lower when using a 3.0-T device (0.96; 95% CI: 0.93-0.97) than when using a 1.0-T device (0.99; 95% CI: 0.96-1.00). Regarding the MR pulse sequences for observing cervical invasion, the three studies 10,16,17 that used DWI alone had higher sensitivity (0.86; 95% CI: 0.71-0.95) than the studies that used DCE-MRI (0.80; 95% CI: 0.65-0.91) or T2-weighted imaging (T2WI) (0.73; 95% CI: 0.64-0.80) alone. The four studies 11,13,14,43 that used DCE-MRI alone presented higher sensitivity (0.80; 95% CI: 0.65-0.91) and specificity (0.96; 95% CI: 0.92-0.98) than those studies that used T2WI alone. T2WI combined with DCE-MRI did not improve diagnostic performance in comparison with DCE-MRI alone. For the depth of cervical invasion, the pooled sensitivity and specificity of MRI were 0.55 (95% CI 0.50-0.61) and 0.95 (95% CI 0.94-0.96) respectively for assessing stromal invasion in endometrial carcinoma.

## Discussion

This meta-analysis demonstrated high pooled specificity of MRI for detecting any cervical infiltration and stromal invasion in patients with endometrial carcinoma. Sensitivity analyses revealed that magnetic field and MR pulse sequences were helpful in explaining the heterogeneity observed for the sensitivity and specificity of MRI for detecting cervical invasion.

The clinical management and the prognosis of endometrial carcinoma are closely related to cervical invasion 4. Using a preoperative technique to detect cervical invasion of endometrial carcinoma may be helpful to reduce the scope of operation, minimize costs, and offer fertility-preserving treatment options for young women without cervical invasion 54. MRI is considered the best non-invasive method for the preoperative staging of endometrial carcinoma 9. In this meta-analysis, MRI showed low sensitivity (0.58) and high specificity (0.95) for detecting cervical invasion. This finding is similar to that in a previous meta-analysis 55. Moreover, further sensitivity analyses of magnetic field strength were performed in our meta-analysis. We found that studies with higher field strength (3.0 T) had higher sensitivity (0.74) than studies with a 1.5-T device (0.60) or 1.0-T device (0.51). Hori et al 25 discovered that 3.0-T imaging improved the tumour signal-to-noise ratio by approximately 12% compared with that of 1.5-T imaging. The main reason for this result is that the signal-to-noise ratio is influenced by the magnetic field strength, with higher fields having a better signal-to-noise ratio. Hence, a 3.0-T device can provide better quality MRI and demonstrate higher pooled sensitivity (0.74) for detecting cervical invasion in endometrial carcinoma. However, there are some problems associated with 3.0-T imaging, particularly of the pelvis, such as a larger susceptibility effect and larger chemical shift 25. These factors may affect the diagnostic accuracy for detecting cervical infiltration in endometrial carcinoma. As a consequence, the pooled specificity was not the highest for the studies using a 3.0-T device (0.96).

T2WI is a conventional MR pulse sequence and one of the best MRI protocols for staging in patients with endometrial carcinoma according to the Updated Guidelines of the European Society of Urogenital Radiology 56. On T2WI, cervical invasion is defined as a mass within the endocervical canal and/or disruption of the normal cervical stroma 25. The normal cervical stroma appears hypointense on T2WI due to its rich fibrous tissue, and endometrial carcinoma appears hyperintense, leading to high contrast resolution 31. Consequently, MRI shows high specificity for detecting cervical invasion. A high specificity means the misdiagnosis rate (false positive rate) is low, and indicates to be good at ruling out cervical invasion. Therefore, if MRI findings do not suggest cervical invasion, that a simple hysterectomy and bilateral salpingo-oophorectomy may be the best surgical option. However, microscopic cervical infiltration may not be observed by using MRI, and only macroscopic cervical invasion could be found, resulting in low sensitivity for detecting cervical invasion in patients with endometrial carcinoma 55. A low sensitivity means the missed diagnosis rate (false negative rate) is high. If we only rely on T2WI to judge the cervical invasion, many patients will be missed diagnosis, so that no radical surgical resection will be performed, which will affect the prognosis of patients. Therefore, DCE-MRI is necessary for preoperatively detecting cervical invasion.

According to a recent meta-analysis, DCE-MRI can help improve the sensitivity and specificity for detecting myometrial invasion 55 because DCE-MRI provides the observer with obvious contrast resolution between the markedly enhanced normal myometrium and the moderately enhanced tumour. On DCE-MRI, cervical invasion is defined as the interruption of the normal cervical epithelium enhancement 16. Moreover, delayed DCE-MRI (4-5 min after the injection) is optimal for the detection of cervical invasion 56. Previous research reported that DCE-MRI improved the detection of cervical infiltration by endometrial carcinoma 11. Our meta-analysis also found that, compared with T2WI, DCE-MRI could improve the sensitivity (0.80) and specificity (0.96) of detection. DCE-MRI is accepted as the state-of-the-art standard for tumour delineation and as one of the best approaches for the local staging of endometrial carcinoma 56. However, it is commonly difficult to assess cervical invasion once endometrial carcinoma spreads to the endocervical canal and begins obliterating the interface between the tumour and the cervix 57. Other MRI functional imaging techniques are needed for accurate preoperative evaluation of cervical infiltration.

DWI is a functional technique of MRI that reflects the diffusivity of the water molecules in tumours. DWI offers potential advantages over DCE-MRI because it does not require contrast administration and it entails a shorter imaging time. Recent evidence suggests that DWI improves the evaluation of the myometrial invasion of endometrial carcinoma because DWI is able to determine malignant lesions as a hyperintense area with excellent tissue contrast 10. To avoid the influence of the T2 shine-through effect, cervical invasion was defined as the appearance of higher signal intensity on high-b-value DWI and low signal intensity on apparent diffusion coefficient (ADC) maps compared with the surrounding normal cervical parenchyma 16,56. This meta-analysis found that studies that used DWI alone had higher sensitivity (0.86) than studies that used DCE-MRI (0.80) or T2WI (0.73) alone. A significant improvement in the sensitivity was also found in DWI compared with that of DCE-MRI and T2WI for detecting cervical invasion in a previous original study 16. The false positive rate may increase when cervical mucus is present because mucus shows a high signal on DWI and a low signal on ADC maps, leading to a decrease in specificity. Furthermore, DWI also has other disadvantages, such as limited spatial resolution and image distortions because of susceptibility artefacts. Thus referring to other MR pulse sequences for an anatomical landmark is warranted. As a result, DWI is now routinely used as an adjunct to T2WI and DCE-MRI 56.

There are some limitations in this meta-analysis. First, due to the lack of sufficient supporting literature, sensitivity analyses of other techniques, such as CO2-volumetric interpolated breath-hold examinations, were not performed. Second, some studies did not report the sequences for observing cervical infiltration. In addition, the number of the included studies was limited. It remains to be determined whether combined DWI and T2WI is superior to DCE-MRI and whether a 3.0-T device combined with DWI or DCE-MRI has higher sensitivity and specificity than other devices. Third, publication bias was present among the included studies. One possible reason was that we excluded relevant studies published in other languages. Other possible reasons were that the sensitivity for detecting cervical invasion was low and some articles with negative results might not have been published.

In conclusion, this meta-analysis shows a low pooled sensitivity and high specificity of MRI for detecting any cervical infiltration and stromal invasion in endometrial carcinoma. The studies with a 3.0-T device demonstrated a higher pooled sensitivity than any other study. The higher the field strength was, the higher the pooled sensitivity. Using DWI alone demonstrated higher pooled sensitivity than using DCE-MRI or T2WI only. Studies that used DCE-MRI alone showed higher sensitivity and specificity than those that used T2WI alone.

## Figures and Tables

**Table 1:** Description of Included Studies

Study	Year	Country	Age (y)	Sample size	Design	Patient recruitment	Blind to reference	Magnetic field	Manufacturer	Sequences	Depth of cervical invasion
Morimura	2000	Japan	U	47	R	U	U	U	U	T2	Any cervix
Seki	2000	Japan	U	39	P	U	Yes	1.5T	Siemens	DCE	Any cervix
Cunha	2001	Portugal	63.2	40	P	U	Yes	1.0T	Philips	T2+DCE	Any cervix
Manfredi	2004	Italy	58.8	37	P	C	Yes	1.5T	GE	T2	Any cervix
Akaeda	2005	Japan	56.8	21	P	U	Yes	1.5T	Siemens	CO2-VIBE	Any cervix
Haider	2006	Canada	56	38	R	U	Yes	1.5T	GE	T2	Any cervix
Nagar	2006	UK	65.5	135	R	C	Yes	1.5T	Siemens	T2	Stroma
Rockall	2007	UK	61	84	R	U	Yes	1.5T	GE	DCE	Stroma
Vasconcelos	2007	Portugal	68.5	101	P	U	Yes	1.0T	Philips	T2+DCE	Any cervix
Cabrita	2008	Portugal	64.6	162	U	U	U	1.5T	U	U	Any cervix
Cicinelli	2008	Italy	67.3	100	U	C	Yes	1.5T	Philips	T2	Any cervix
Sanjuan	2008	Spain	U	72	R	C	U	1.0T	Siemens	T2+DCE	Any cervix
Savelli	2008	Italy	63	74	P	C	Yes	U	U	T2	Any cervix
Hori	2009	Japan	58.7	30	P	C	Yes	3.0T	GE	T2	Any cervix
Undurraga	2009	Switzerland	69.5	108	R	C	Yes	1.5T	U	T2+CE	Stroma
Celik	2010	Turkey	58.9	64	P	C	Yes	1.5T	Siemens	U	Any cervix
Emlik	2010	Turkey	U	53	P	C	Yes	1.5T	Siemens	DCE	Any cervix
Duncan	2012	UK	U	748	U	U	U	U	U	U	Stroma
Haldorsen	2012	Norway	66	146	P	U	Yes	1.5T	Siemens	U	Stroma
Tong	2012	China	52	168	R	C	U	1.5T	GE	T2+DCE	Stroma
Zamani	2012	Iran	53.3	54	U	U	Yes	1.5T	U	U	Stroma
Aly	2013	Egypt	59	40	U	U	Yes	1.5T	GE	DCE	Stroma
Antonsen	2013	Denmark	65	226	P	C	Yes	1.5T	Philips	U	Any cervix
Foti	2013	Italy	62	20	P	C	Yes	1.5T	GE	T2	Any cervix
Hahn	2013	Korea	53.1	131	R	U	Yes	1.5T	Philips	U	Stroma
Hori	2013	Japan	57.6	71	P	C	Yes	3.0T	Philips	T2+DWI	Stroma
Kitajima	2013	Japan	62.4	30	R	U	Yes	1.5T	GE	U	Stroma
Gitte	2013	Denmark	U	143	P	U	Yes	1.5T	GE	U	Any cervix
Koplay	2014	Turkey	58	58	P	C	Yes	1.5T	Siemens	DWI	Any cervix
Teng	2015	China	57.9	167	R	U	Yes	1.5T	GE	T2+DCE	Any cervix
Yin	2015	China	54.6	98	R	U	Yes	3.0T	U	T2+DCE	Any cervix
Zamani	2015	Iran	U	68	P	U	Yes	U	U	U	Stroma
Angioli	2016	Italy	53	41	P	U	Yes	1.5T	GE	DWI	Any cervix
Chan	2016	China	55.2	90	R	U	Yes	1.5T	Siemens	T2+DCE	Stroma
Shrivastava	2016	India	52.8	36	R	U	Yes	1.5T	Philips	U	Stroma
Lin	2017	China	56	83	U	C	Yes	3.0T	Siemens	DWI	Stroma
Rahmani	2018	Iran	U	27	P	U	Yes	3.0T	Siemens	U	Any cervix
Xu	2018	China	51.89	88	R	U	U	U	U	U	Any cervix
Yildirim	2018	Turkey	61.1	40	P	U	Yes	1.5T	Philips	U	Any cervix
Ytre-Hauge	2018	Norway	67	178	P	C	Yes	1.5T	Siemens	U	Stroma
Goel	2019	India	60.2	58	P	U	Yes	1.5T	GE	T2+DCE	Any cervix
Yang	2019	China	54.1	182	R	U	Yes	3.0T	GE	T2	Any cervix

U, unknown; P, prospective; R, retrospective; C, consecutive; CE, contrast-enhanced MRI; DCE, dynamic contrast-enhanced MRI; DWI, diffusion weighted imaging; CO2-VIBE, CO_2_-volumetric interpolated breathhold examination.

**Table 2:** The Results of Meta-Regression of MRI

Variable	Coefficient	Standard error	*P* value	Diagnostic odd ratio	95 % CI
Age	0.402	0.2274	0.0850	1.49	(0.94-2.37)
Design	-0.082	0.2363	0.7296	0.92	(0.57-1.49)
Blind to reference	0.444	0.4159	0.2926	1.56	(0.67-3.61)
Magnetic field	0.383	0.2181	0.0865	1.47	(0.94-2.28)
Sequences	0.109	0.0660	0.1080	1.11	(0.98-1.27)
Depth of cervical invasion	-0.043	0.3423	0.8996	0.96	(0.48-1.91)
Interval between MRI and pathology	-0.309	0.3274	0.3518	0.73	(0.38-1.42)

CI, confidence interval.

**Table 3:** Sensitivity Analyses Performed for Subgroups of Studies

Analysis	Number of studies	Sensitivity	Specificity	PLR	NLR	DOR
Overall	42	0.58 (0.55-0.62)	0.95 (0.94-0.95)	**9.37 (7.78-11.28)**	0.43 (0.36-0.51)	**29.68 (21.16-41.63)**
**Age (y)**						
≥ 60	15	0.51 (0.45-0.56)	0.93 (0.92-0.95)	**6.73 (5.18-8.74)**	0.54 (0.44-0.67)	**15.54 (9.49-25.45)**
< 60	19	0.72 (0.66-0.78)	0.94 (0.93-0.96)	**10.95 (8.61-13.94)**	0.27 (0.18-0.42)	**58.71 (37.51-91.89)**
**Design**						
Prospective	21	0.58 (0.52-0.64)	0.94 (0.92-0.95)	**7.96 (5.93-10.68)**	0.43 (0.33-0.56)	**26.78 (15.43-46.50)**
Retrospective	15	0.64 (0.58-0.70)	0.94 (0.93-0.95)	**9.62 (7.53-12.30)**	0.38 (0.26-0.54)	**35.25 (23.09-53.81)**
**Blind to reference**						
Yes	36	0.61 (0.57-0.65)	0.94 (0.93-0.95)	**9.81 (7.82-12.30)**	0.39 (0.32-0.49)	**34.44 (23.00-51.57)**
Unknown	6	0.51 (0.44-0.59)	0.95 (0.93-0.96)	**8.76 (6.21-12.35)**	0.53 (0.39-0.72)	**19.45 (11.01-34.34)**
**Magnetic field**						
3.0T	6	**0.74 (0.60-0.84)**	0.96 (0.93-0.97)	**16.22 (8.69-30.25)**	**0.33 (0.19-0.58)**	**68.56 (28.18-166.78)**
1.5T	28	0.60 (0.56-0.65)	**0.93 (0.92-0.94)**	**8.15 (6.61-10.04)**	0.40 (0.31-0.51)	**27.08 (17.60-41.66)**
1.0T	3	**0.51 (0.37-0.65)**	**0.99 (0.96-1.00)**	**19.99 (5.73-69.76)**	**0.54 (0.39-0.76)**	**39.81 (9.15-173.19)**
**MR pulse sequences**						
DWI	3	**0.86 (0.71-0.95)**	**0.92 (0.87-0.96)**	**10.18 (4.97-20.86)**	**0.16 (0.07-0.37)**	**61.42 (19.65-191.93)**
DCE	4	**0.80 (0.65-0.91)**	**0.96 (0.92-0.98)**	**17.65(8.10-38.48)**	0.21 (0.07-0.61)	**78.46 (24.60-250.18)**
T2	9	**0.73 (0.64-0.80)**	**0.92 (0.89-0.94)**	**8.34 (6.14-11.33)**	0.33 (0.21-0.51)	**34.57 (19.48-61.35)**
T2+DCE	9	0.60 (0.52-0.67)	0.96 (0.94-0.97)	**14.35 (7.78-26.46)**	0.45 (0.30-0.67)	**42.38 (20.56-87.35)**
**Depth of cervical invasion**						
Stromal invasion	16	0.55 (0.50-0.61)	0.95 (0.94-0.96)	**9.29 (7.01-12.30)**	0.46 (0.36-0.59)	**25.98 (16.52-40.85)**
Any cervical invasion	26	0.61 (0.56-0.66)	0.94 (0.93-0.95)	**9.53 (7.38-12.32)**	0.39 (0.30-0.50)	**33.69 (20.43-55.56)**
**Interval between MRI and pathology**						
Appropriate	24	0.59 (0.54-0.63)	0.94 (0.93-0.95)	**9.10 (7.00-11.83)**	0.42 (0.32-0.54)	**30.02 (17.73-50.80)**
Unknown	18	0.58 (0.53-0.64)	0.95 (0.94-0.96)	**9.86 (7.68-12.65)**	0.43 (0.34-0.54)	**28.66 (20.11-40.86)**

PLR, positive likelihood ratio; NLR, negative likelihood ratio; DOR, diagnostic odds ratios; DWI, diffusion weighted imaging; DCE, dynamic contrast-enhanced MRI. Data in parentheses are 95% confidence interval. Bold fonts, indicating *I^2^* values < 50.0%.

## Abbreviations

DWI: diffusion-weighted imaging
DCE-MRI: dynamic contrast-enhanced MRI
T2WI: T2-weighted image
ADC: apparent diffusion coefficient
sROC: summary receiver-operating characteristics
CI: confidence interval
PRISMA-DTA: Preferred Reporting Items for Systematic Reviews-Diagnostic Test Accuracy
QUADAS-2: Quality Assessment of Diagnostic Accuracy Studies-2
